# CPEB3 deficiency in mice affect ovarian follicle development and causes premature ovarian insufficiency

**DOI:** 10.1038/s41419-021-04374-4

**Published:** 2021-12-20

**Authors:** Fang E., He Zhang, Wanli Yin, Chongyang Wang, Yuanli Liu, Yanze Li, Linlin Wang, Yue Wu, Runze Zhang, Chendan Zou, Tianjun Song, Cedric Matunda, Chaoxia Zou, Xu Gao

**Affiliations:** 1 Department of Biochemistry and Molecular Biology, Harbin Medical University, Harbin, Heilongjiang 150081 China; 2 Public Laboratory, Tianjin Medical University Cancer Institute and Hospital, National Clinical Research Center for Cancer, Tianjin, 300060 China; 3 Department of General Surgery, Heilongjiang Provincial Hospital Affiliated to Harbin Institute of Technology, Harbin, Heilongjiang 150000 China; 4 Department of Biochemistry & Biotechnology, The Technical University of Kenya, Nairobi, Kenya; 5 Translational Medicine Research and Cooperation Center of Northern China, Heilongjiang Academy of Medicine Sciences, Harbin, Heilongjiang 150081 China; 6 Key Laboratory of Cardiovascular Medicine Research of Harbin Medical University, Ministry of Education, Harbin, Heilongjiang 150081 China; 7 Key laboratory of preservation of human genetic resources and disease control in China (Harbin Medical University), Ministry of Education, Harbin, Heilongjiang China

**Keywords:** RNA-binding proteins, Infertility

## Abstract

Premature ovarian insufficiency (POI) is a heterogeneous and multifactorial disorder. In recent years, there has been an increasing interest in research on the pathogenesis and treatment of POI, owing to the implementation of the second-child policy in China. Cytoplasmic polyadenylation element-binding protein 3 (CPEB3) is an RNA-binding protein that can bind to specific RNA sequences. CPEB3 can bind to and affect the expression, cellular location, and stability of target RNAs. *Cpeb3* is highly expressed in the ovary; however, its functions remain unknown. In this study, *Cpeb3*-mutant mice were used to characterize the physiological functions of CPEB3. *Cpeb3*-mutant female mice manifested signs of gradual loss of ovarian follicles, ovarian follicle development arrest, increased follicle atresia, and subfertility with a phenotype analogous to POI in women. Further analysis showed that granulosa cell proliferation was inhibited and apoptosis was markedly increased in *Cpeb3*-mutant ovaries. In addition, the expression of *Gdf9*, a potential target of CPEB3, was decreased in *Cpeb3*-mutant ovaries and oocytes. Altogether, these results reveal that CPEB3 is essential for ovarian follicle development and female fertility as it regulates the expression of *Gdf9* in oocytes, disruption of which leads to impaired ovarian follicle development and POI.

## Introduction

Primary ovarian insufficiency (POI) is a subclass of ovarian dysfunction in which the cause of the disease is within the ovary [1]. POI is defined as a loss of ovarian function before the age of 40 and it is diagnosed by elevated serum follicle-stimulating hormone (FSH) levels (>40 IU/L), which accounts for one major cause of female infertility [2]. POI might also result from genetic defects, chemotherapy, radiotherapy, or surgery [3]. On the molecular level, multiple gene mutations can cause POI [4]. The ovarian follicle, which consists of an oocyte (egg) surrounded by one or more layers of granulosa cells, is the functional content of the ovary. Follicle development stages include the primordial follicle, primary follicle, secondary follicle, and antral follicle [5]. Ovarian follicle development is precisely regulated by a sequence of autocrine and paracrine factors [6]. Abnormal development of follicles leads to subfertility and POI.

The CPEB family of proteins (CPEB1–4) are all sequence-specific RNA-binding proteins. Previous studies have confirmed that CPEB family proteins can regulate the expression of target mRNA by changing the length of the polyadenylic acid elongation of the target mRNA [7, 8]. CPEB family proteins can recognize the classical cytoplasmic polyadenylation element (sequence: UUUUUAU) in the untranslated region (UTR) of the target mRNA [9]. Cytoplasmic polyadenylation element-binding protein 3 (CPEB3) is an RNA-binding protein, highly expressed in brain, heart, reproductive system, and liver. CPEB3 can bind to and affect the expression, cell location, and stability of the target mRNA. Several studies have established that CPEB3 can affect the nervous system by regulating the translation of *Stat5b* [10] and *GluR2* [11] mRNA. Our previous study showed that CPEB3 could inhibit the metastasis of hepatocellular carcinoma (HCC) by inhibiting the expression of MTDH [12]. CPEB3 inhibits HCC progression by regulating the expression of EGFR [13] and can inhibit the metastasis of colorectal cancer by regulating the IL-6/STAT3-signaling pathway [14]. In our previous study, we established the *Cpeb3*-mutant mice and unexpectedly found that *Cpeb3* mutation caused subfertility in female mice. Several studies have reported that CPEB1 [15] and CPEB4 [16] have an important role in the development of oocytes. Therefore, this research attempts to examine the emerging role of CPEB3 in follicle development and POI.

The cellular and molecular mechanisms that determine the developmental fate of ovarian follicles are still poorly understood. The cytoplasmic polyadenylation element-binding (CPEB) proteins are sequence-specific mRNA binding proteins that control translation in development, health, and disease. Masked mRNA refers to dormant transcripts in the oocyte that are later translated during the completion of the meiotic divisions or early embryo development [9]. The study of masked mRNA in developing oocytes has provided critical insights into the mechanisms of translational regulation. GDF9 is required for ovarian folliculogenesis [17] and we found that the phenotype of *Cpeb3*-mutant female mice is analogous to *Gdf9*-mutant mice. In this context, we investigated whether CPEB3 is involved in regulating follicular development and atresia. We systematically examined the development of the ovarian follicles in *Cpeb3*-mutant mice and specifically focused on the correlation between follicular atresia and granulosa cell growth and death.

## Results

### *Cpeb3-*mutant female mice display impaired fertility

We previously reported that *Cpeb3*-mutant mice were generated by the CRISPR/Cas9 method [12] (Supplementary Fig. S1A, B, and C), followed by genotype identification through DNA sequencing and western blotting (Supplementary Fig. S1D, E). The first set of analyses examined the impact of *Cpeb3* mutation in the fertility of female mice, 3-month-old WT and *Cpeb3*-mutant female mice were mated with 2–3-month-old WT males with proven fecundity, for ~6 months. As shown in Table 1, the total number of pups delivered by *Cpeb3*-mutant mice was less than a quarter of WT females (73/334), particularly, a fewer number of total litters (13/41) and an average number of pups per litter in the *Cpeb3*-mutant group (5.615/8.146). These results suggest *Cpeb3*-mutant female mice display impaired fertility compared with WT female mice.

**Table 1:** *Cpeb3* mutation females were subfertile.

Genotype	Total females	Pregnant females	Pregnant ratio	Total no. of pups	Total litters	Average no. of pups/ litter
WT	9	9	100%	334	41	8.146 ± 1.805
*Cpeb3* mut	9	5	55.5%	73	13	5.615 ± 1.636^****^

WT and *Cpeb3-*mutant female mice of 3-month-old were mated with WT 2–3-month-old C57BL/6 J male mice that had proven normal fecundity until the female mice were 9-month-old. Female mice with vaginal plug and later visibly growing abdomen were included in this assessment. The numbers of pups per litter or pups (both alive and dead) were assessed. Data were represented as the mean ± SD. ****P < 0.0001.

### *Cpeb3* mutation reduces ovarian size and weight

**Fig. 1:**
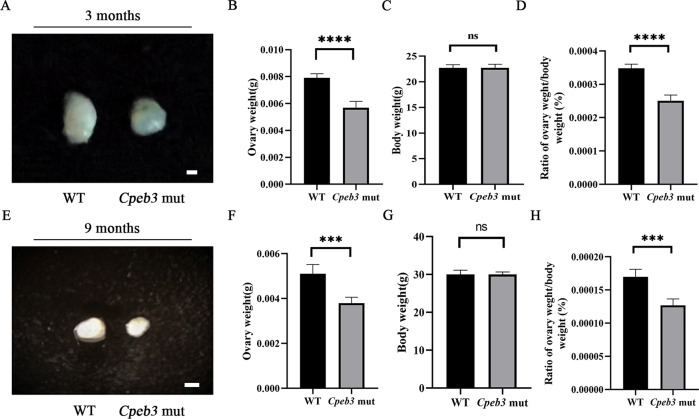
*Cpeb3* mutation causes ovarian atrophy in mice. The representative appearance of 3- and 9-month-old ovaries (**A** and **E**). Five ovaries from each group were measured and weighed. Column charts comparing the ovary weight (**B** and **F**), body weight (**C** and **G**), and the ratio of ovary/body weight (**D** and **H**) among the two groups of mice. Data were represented as the mean ± SD, *n* = 5. ***P < 0.001, ****P < 0.0001, ns. *P* > 0.05.

To explore the reason for *Cpeb3* mutation, leading to female mice subfertility, the morphology of ovaries from WT and *Cpeb3*-mutant mice was observed. The size of ovaries from adult *Cpeb3*-mutant female mice (3- and 9-month-old) was smaller than those from WT female mice (Fig. 1A and E). In addition, the quantitation analysis of the ovary weight showed that *Cpeb3*-mutant mouse ovaries were dramatically lighter compared with WT mouse ovaries, suggesting that mutation of *Cpeb3* impairs ovarian development. The average weight of 3-month-old WT and *Cpeb3*-mutant ovaries was 0.0079 ± 0.00032 g and 0.0057 ± 0.00046 g, respectively (Fig. 1B). The average weight of 9-month-old WT and *Cpeb3*-mutant ovaries was 0.0051 ± 0.00042 g and 0.0038 ± 0.00026 g, respectively (Fig. 1F). Meanwhile, no difference in body weight between WT and *Cpeb3*-mutant female mice was observed. The average body weight of 3-month-old WT and *Cpeb3*-mutant mice was 22.72 ± 0.6221 g and 22.74 ± 0.6877 g, respectively (Fig. 1C). The average body weight of 9-month-old WT and *Cpeb3*-mutant mice was 30.02 ± 1.145 g and 30.00 ± 0.6782 g, respectively (Fig. 1G). The ratio of ovary/body weight is lower in *Cpeb3*-mutant female mice compared with WT female mice (Fig. 1D and H). Overall, these results indicate that *Cpeb3* mutation reduces ovarian size and weight.

### Progressive loss of ovarian follicles in *Cpeb3*-mutant mice ovaries

**Fig. 2:**
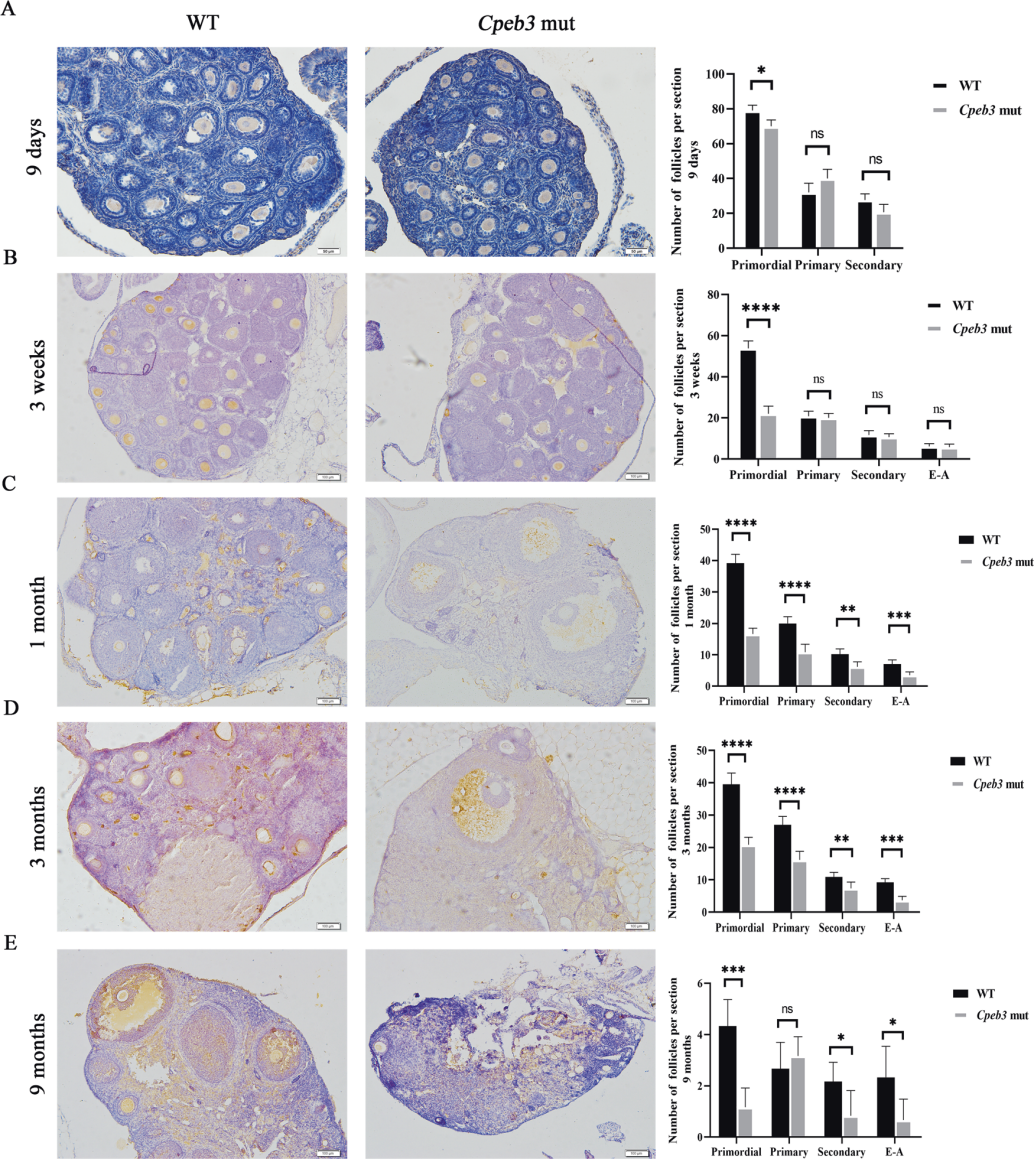
*Cpeb3* mutation decreased the number of follicles in different ages of mice. Ovarian morphologies of 9 days, 3-week, 1-, 3-, and 9-month-old WT and *Cpeb3*-mutant mice. MVH/DDX4 antibody was applied in IHC method to manifest oocytes for easier quantification of follicle numbers. At least three mice of each genotype for each time point were measured. Abbreviations: early antral and antral follicle (**E**–**A**). **A**–**E** Column charts comparing the follicle numbers in 9 days, 3-week-old, 1-month-old, 3-month-old, and 9-month-old female mice per section. Scale bar = 50 μm in **A** and 100 μm in **B**–**E**. Data were represented as the mean ± SD, *n* = 3 in **A** and six in **B**–**E**. *P < 0.05, **P < 0.01, ***P < 0.001, ****P < 0.0001, ns. *P* > 0.05.

CPEB3 protein expression patterns in the ovaries were examined using immunohistochemistry (IHC) staining. The results revealed that CPEB3 was mainly expressed in the oocyte, especially in primordial, primary, and secondary follicles (Supplementary Fig. S2A). To understand how *Cpeb3* mutation impairs female fertility, we compared the follicle numbers of ovaries at different ages. MVH/DDX4 antibody was applied in IHC method to manifest oocytes for easier quantification of follicle numbers. At PD9, the number of primordial and secondary follicles was slightly reduced in *Cpeb3*-mutant mice. However, the number of primary follicles was slightly increased in *Cpeb3*-mutant mice (WT group: primordial follicle = 79.000 ± 3.000, primary follicle = 32.000 ± 5.292, secondary follicle = 27.667 ± 3.512, *Cpeb3*-mutant group: primordial follicle = 70.000 ± 3.606, primary follicle = 40.000 ± 5.292, secondary follicle = 20.667 ± 4.509, *n* = 3. Fig. 2A). The number of primordial follicles was reduced in 3-week-old *Cpeb3*-mutant female mice compared with WT female mice, and slightly reduced in the primary, secondary and antral follicles (WT group: primordial follicle = 53.833 ± 3.656, primary follicle = 20.833 ± 2.483, secondary follicle = 11.667 ± 2.160, E-A follicle = 6.167 ± 1.329, *Cpeb3*-mutant group: primordial follicle = 22.000 ± 3.742, primary follicle = 20.000 ± 2.191, secondary follicle = 10.667 ± 1.633, E-A follicle = 5.833 ± 1.472, *n* = 6. Fig. 2B). The numbers of primordial, primary, secondary and antral follicles were significantly decreased in 1-month-old *Cpeb3*-mutant female mice compared with WT female mice (WT group: primordial follicle = 39.167 ± 2.858, primary follicle = 20.000 ± 2.191, secondary follicle = 10.167 ± 1.722, E-A follicle = 7.000 ± 1.414, *Cpeb3*-mutant group: primordial follicle = 16.667 ± 1.862, primary follicle = 10.883 ± 2.563, secondary follicle = 6.167 ± 1.602, E-A follicle = 3.500 ± 1.049, *n* = 6. Fig. 2C). The numbers of all stages of follicles were significantly decreased in 3-month-old *Cpeb3*-mutant female mice compared with WT female mice (WT group: primordial follicle = 39.500 ± 3.507, primary follicle = 27 ± 2.608, secondary follicle = 10.833 ± 1.472, E-A follicle = 9.167 ± 1.169, *Cpeb3*-mutant group: primordial follicle = 20.833 ± 2.317, primary follicle = 16.167 ± 2.639, secondary follicle = 7.333 ± 1.966, E-A follicle = 3.667 ± 1.211, *n* = 6. Fig. 2D). Interestingly, there was a decrease in the number of primordial, secondary and antral follicles in 9-month-old *Cpeb3*-mutant female mice, but the number of primary follicles were insignificantly increased compared with WT female mice (WT group: primordial follicle = 4.333 ± 1.033, primary follicle = 2.667 ± 1.033, secondary follicle = 0.3073 ± 0.753, E-A follicle = 2.333 ± 1.211, *Cpeb3*-mutant group: primordial follicle = 1.167 ± 0.753, primary follicle = 3.167 ± 0.753, secondary follicle = 0.833 ± 0.983, E-A follicle = 0.667 ± 0.816, *n* = 6. Fig. 2E). Subsequently, the morphology of ovaries of WT and *Cpeb3*-mutant female mice were compared by HE staining (Supplementary Fig. S2B). There were more degenerating oocytes and impeded developing follicles in *Cpeb3*-mutant female mice (Supplementary Fig. S2C). In summary, these results showed that there is a progressive loss of ovarian follicles in *Cpeb3*-mutant ovaries.

### *Cpeb3* mutation enhances follicular atresia

**Fig. 3:**
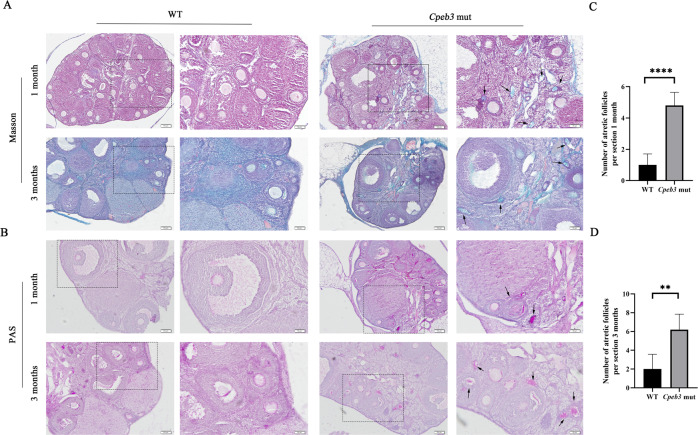
Ovarian sections stained with PAS and Masson. **A** Representative micrographs of 1- and 3-month-old WT and *Cpeb3*-mutant ovarian sections stained with Masson’s trichrome stain. **B** Histological sections of 1- and 3-month-old WT and *Cpeb3*-mutant ovaries were stained with periodic acid Schiff (PAS) staining. **C** Quantification of atretic follicles of 1-month-old WT and *Cpeb3*-mutant ovaries per section. **D** Quantification of atretic follicles of 3-month-old WT and *Cpeb3*-mutant ovaries per section. Arrows indicating the atretic follicles. Scale bar = 100 μm and 50 μm in **A**, **B**. Data were represented as the mean ± SD, *n* = 5. **P < 0.01, ****P < 0.0001.

Histological sections of 1- and 3-month-old WT and *Cpeb3*-mutant ovaries were stained with Masson and Periodic Acid Schiff (PAS) staining to reveal the extent of follicle atresia in these ovaries (Fig. 3A, B). The high magnification of the zona pellucida shows its distinct contraction in the atretic follicles. The irregular shape of the zona pellucida is a symbol of atretic follicles. The number of atretic follicles was counted in WT and *Cpeb3*-mutant ovaries. The results showed that the number of atretic follicles increased in 1-month-old *Cpeb3*-mutant ovaries (WT group: 1.0 ± 0.7071, *Cpeb3*-mutant group: 4.800 ± 0.8367, *n* = 5. Fig. 3C). Similarly, the number of atretic follicles increased in 3-month-old *Cpeb3*-mutant ovaries (WT group: 2.0 ± 1.581, *Cpeb3*-mutant group: 6.200 ± 1.634, *n* = 5. Fig. 3D). These results indicate that *Cpeb3* mutation enhances follicle atresia in female mice.

### *Cpeb3* mutation increases apoptosis and decreases the proliferation of granulosa cells

**Fig. 4:**
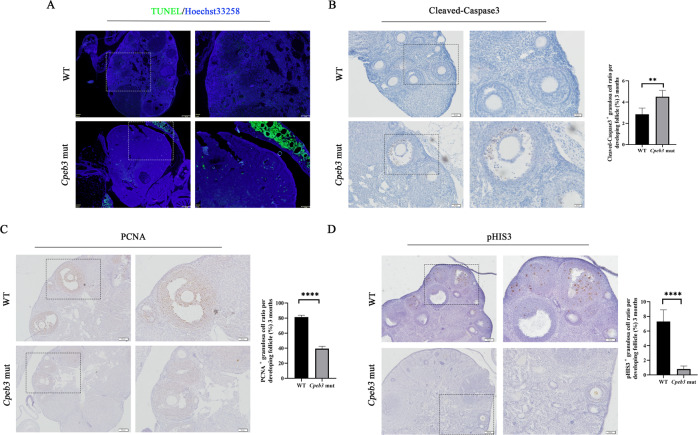
*Cpeb3* mutation affects granulosa cells apoptosis and proliferation. **A** Apoptosis of ovaries was assessed by TUNEL assay at 3-month-old ovaries. **B** Representative micrographs of follicles stained for Cleaved-Capase3 at 3-month-old ovaries. **C**, **D** Ovarian sections stained for PCNA and pHIS3 at 3-month-old ovaries. The number of results was accounted for per developing follicle. Scale bar = 161 μm and 64 μm in **A**; 100 μm and 50 μm in **B**–**D**. Data were represented as the mean ± SD ****P < 0.0001.

We explored the extent of apoptosis in WT and *Cpeb3*-mutant ovaries by TUNEL and IHC staining using Cleaved-Caspase3 antibody. Compared with WT ovaries, more TUNEL positive granulosa cells were noticed in 3-month-old *Cpeb3*-mutant ovaries (Fig. 4A). To evaluate the apoptosis of the ovaries in WT and *Cpeb3*-mutant mice, IHC staining for Cleaved-Caspase3 was performed. The results revealed that more Cleaved-Caspase3 positive granulosa cells were found in *Cpeb3*-mutant ovaries. (1-month, WT group: 1.415% ± 0.2848%, *Cpeb3*-mutant group: 2.399% ± 0.6102%, *n* = 5. Supplementary Fig. S3A; 3-month, WT group: 2.845% ± 0.5921%, *Cpeb3*-mutant group: 4.495% ± 0.6058%, *n* = 5. Fig. 4B). Besides, autophagy is another mechanism to cause follicle atresia. Autophagy levels in WT and *Cpeb3*-mutant ovaries were examined using IHC staining for Beclin1; however, there were no differences between the two groups (Supplementary Fig. S3B). Subsequently, the relationship between CPEB3 and granulosa cell proliferation was investigated. Ovary sections of 1- and 3-month-old WT and *Cpeb3*-mutant ovaries were IHC stained with proliferating cell nuclear antigen (PCNA) and pHIS3 antibody. The examination revealed that the number of PCNA-positive granulosa cells in both 1- and 3-month-old *Cpeb3*-mutant ovaries were significantly reduced compared with WT ovaries (1-month, WT group: 70.72% ± 1.789%, *Cpeb3*-mutant group:18.00% ± 2.454%, *n* = 6. Supplementary Fig. S3C; 3-month, WT group: 81.37% ± 2.308%, *Cpeb3*-mutant group: 39.40% ± 3.153%, *n* = 6. Fig. 4C). Furthermore, pHIS3 positive granulosa cells was significantly reduced in both 1- and 3-month-old *Cpeb3*-mutant ovaries compared with WT ovaries (1-month, WT group: 5.767% ± 0.8311%, *Cpeb3*-mutant group: 1.717% ± 0.3764%, *n* = 6. Supplementary Fig. S3D; 3-month, WT group: 7.300% ± 1.592%, *Cpeb3*-mutant group: 0.800% ± 0.4183%, *n* = 6. Fig. 4D). These results indicate that the loss of ovarian follicles in *Cpeb3*-mutant female mice is associated with the apoptosis and proliferation of granulosa cells.

### *Cpeb3* mutation causes POI in female mice

**Fig. 5:**
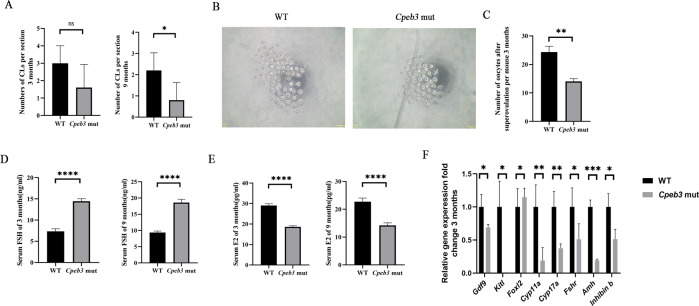
*Cpeb3* mutation induces POI in mice. **A** Quantification of CL in 3- and 9-month-old WT and *Cpeb3*-mutant ovaries per section. **B** Representative images of in vivo matured eggs from WT and *Cpeb3*-mutant mice. **C** The number of eggs obtained from WT and *Cpeb3*-mutant mice per mice. **D** Serum levels of FSH in 3- and 9-month-old female mice. **E** Serum levels of E2 in 3- and 9-month-old female mice. **F** POI relative mRNA levels were performed by qRT-PCR in ovaries from 3-month-old female mice. Scale bar = 153 μm in **B**. Data were represented as the mean ± SD, *n* = 5 in **A**, three in **B** and **F**, six in **D** and **E**. *P < 0.05, **P < 0.01, ****P < 0.0001, ns. *P* > 0.05.

As we observed the loss of follicles in *Cpeb3*-mutant ovaries, the number of corpus luteum (CL) was counted in WT and *Cpeb3*-mutant ovaries. The number of CL were decreased in 3- and 9-month-old *Cpeb3*-mutant mice (3-month, WT group: 3 ± 1.000, *Cpeb3*-mutant group: 1.6 ± 1.342, *n* = 5; 9-month, WT group: 2.2 ± 0.8367, *Cpeb3*-mutant group: 0.8 ± 0.8367, *n* = 5. Fig. 5A). In addition, 3-month-old female mice were superovulated to collect in vivo matured eggs. Compared with WT female mice, the number of eggs retrieved from *Cpeb3*-mutant mice was significantly reduced (WT group: 24.33 ± 2.082, Cpeb3-mutant group: 14 ± 1.000, *n* = 3. Fig. 5B, C). We thereafter, measured serum hormone levels in female mice aged ~3 and 9 months. The levels of FSH were elevated in *Cpeb3*-mutant female mice (3-month, WT group: 7.362 ± 0.5933 ng/ml, *Cpeb3*-mutant group: 14.44 ± 0.6191 ng/ml, *n* = 6; 9-month, WT group: 9.338 ± 0.4920 ng/ml, *Cpeb3*-mutant group: 18.57 ± 1.018 ng/ml, *n* = 6. Fig. 5D), whereas estradiol (E2) was significantly decreased (3-month, WT group: 29.01 ± 0.8776 pg/ml, *Cpeb3*-mutant group: 18.59 ± 0.5437 pg/ml, *n* = 6; 9-month, WT group: 22.74 ± 1.296 pg/ml, *Cpeb3*-mutant group: 14.22 ± 0.9161 pg/ml, *n* = 6. Fig. 5E). These phenotypes are analogous to POI of human patients. qRT-PCR was used to examine the expression of genes known to be associated with POI in 3-month-old WT and *Cpeb3*-mutant ovaries. The results demonstrated that *Gdf9*, *Kitl*, *Cyp11a*, *Cyp17a*, *Fshr*, *Amh*, and *inhibin B* expression were significantly altered in *Cpeb3*-mutant ovaries compared with WT ovaries (Fig. 5F). These data indicate *Cpeb3* mutation may be one of the potential pathological causes of POI.

### *Cpeb3* mutation impaired the expression of the *Gdf9* in oocytes

**Fig. 6:**
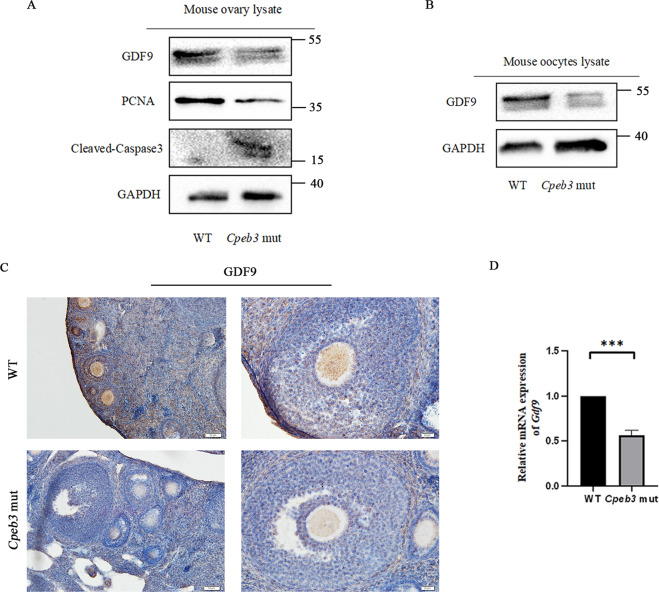
*Cpeb3* mutation causes POI in mice by downregulation of the oocyte-derived factor, *Gdf9*. **A** Western blot analysis of GAPDH, GDF9, PCNA, and Cleaved-Caspase3 protein expression in 3-month-old WT and *Cpeb3*-mutant ovaries. GAPDH was used as the internal control. **B** Western blot analysis of GDF9 and GAPDH in WT and *Cpeb3*-mutant oocytes. GAPDH was used as the internal control. **C** Ovarian sections stained for GDF9 at 3-month-old WT and *Cpeb3*-mutant ovaries. **D** *Gdf9* mRNA levels were performed by qRT-PCR in WT and *Cpeb3-*mutant oocytes. *Gapdh* was used as the internal control. Scale bar = 50 μm and 25 μm in **C**. ***P < 0.001.

The phenotype of *Cpeb3*-mutant ovaries is analogous to *Gdf9*-mutant mice. To investigate the mechanism underlying *Cpeb3* mutation that caused follicle loss and the impediment of follicle development. We firstly investigated the expression of *Gdf9* in *Cpeb3*-mutant ovaries and the protein level of GDF9 was determined by western blotting. Results demonstrated that GDF9 expression was decreased in 3-month-old *Cpeb3*-mutant ovaries compared with WT ovaries (Fig. 6A). The protein levels of PCNA and Cleaved-Caspase3 also altered in *Cpeb3*-mutant ovaries (Fig. 6A). IHC staining using GDF9 antibody confirmed that the protein level of GDF9 was decreased in *Cpeb3*-mutant ovaries compared with WT ovaries (Fig. 6C). Also, GDF9 protein levels were detected in oocytes by western blotting. Consistent with our results in ovaries, the GDF9 protein levels are decreased in *Cpeb3*-mutant oocytes compared with WT oocytes (Fig. 6B). Besides, we also extracted mRNA from the oocytes of WT and *Cpeb3*-mutant mice for qRT-PCR. Results showed that the mRNA level of *Gdf9* is decreased in *Cpeb3*-mutant oocytes compared with WT oocytes (Fig. 6D). Altogether, these results suggest that *Cpeb3* mutation impaired the expression of the *Gdf9* in oocytes.

## Discussion

CPEB3 belongs to the family of CPEBs, which plays a critical role in regulating mRNA expression and maintenance [18, 19]. CPEB3 was reported as a tumor suppressor in our previous work [12]. In this study, we unexpectedly found that *Cpeb3* mutation caused subfertility in female mice. CPEB3 is highly expressed in the reproductive system, especially in the oocytes of primary and secondary follicles. We examined and compared the morphology of WT and *Cpeb3*-mutant ovaries and the average weight and size of the *Cpeb3*-mutant ovaries were significantly smaller than WT ovaries, even though there was no significant difference between the overall weights of these mice. A significant reduction of follicles and an increase of atretic follicles in *Cpeb3*-mutant mice was noticed. These results suggest that CPEB3 might play a specific role in ovarian follicle development. Combined with the fact that there is a reduction in the number of ovulations after superovulation and a change in serum levels of FSH and E2 in *Cpeb3*-mutant mice, these phenotypes are analogous to the POI of human patients. Moreover, the loss of follicles was observed in 3-week-old (before puberty) *Cpeb3*-mutant female mice. This suggests that CPEB3 is necessary for ovarian follicle development and female fecundity and the damage of ovarian function caused by *Cpeb3* mutation is gonadotropin independent.

POI or premature ovarian failure with defects in follicular development in the ovary has become the main cause of female infertility, which affects ~1% of women under 40 years old [20]. The normal length of reproductive life and menopausal age in women is determined by the reserve of primordial follicles in the ovaries [21]. *Cpeb3* mutation in mice causes both premature depletion of the primordial follicle pool and the gradual loss of all stages of ovarian follicles, manifesting a typical etiology of POI in humans.

Folliculogenesis involves the activation of a small number of primordial follicles, which then develop and pass through the primary, secondary, and antral follicle stages. Only a few of these mature follicles are ovulated, whereas the majority normally undergoes atresia in ovaries [22, 23]. Hence, we investigated whether the reduction in the number of follicles was attributed to reduced cell proliferation and enhanced apoptosis of granulosa cells. In addition, there were no differences in autophagy levels between the two groups. Moreover, there are more impeded developing follicles in *Cpeb3*-mutant ovaries, where most follicles are impeded in the primary or secondary stages. It is analogous to the phenotype of *Gdf9*-mutant female mice.

The genetic disorder is a major cause of POI and there are about 1–4% of genetic defects in POI patients caused by *Gdf9* mutation [24, 25]. GDF9, a secreted protein from the oocyte in ovary follicles, plays a major role in follicle development [26, 27]. GDF9 can affect the proliferation, differentiation, steroid hormone synthesis, apoptosis, and cumulus expansion of granulosa cells [28–31]. We found that mRNA of *Gdf9* has two CPE in its UTR region. Our previous RIP-seq analysis showed that mRNA of *Gdf9* is a potential target of CPEB3 [12]. To elucidate if CPEB3 affects *Gdf9* expression, we performed qRT-PCR and western blotting analyses. Our results showed that *Cpeb3* mutation decreased *Gdf9* expression in mRNA and protein levels.

**Fig. 7:**
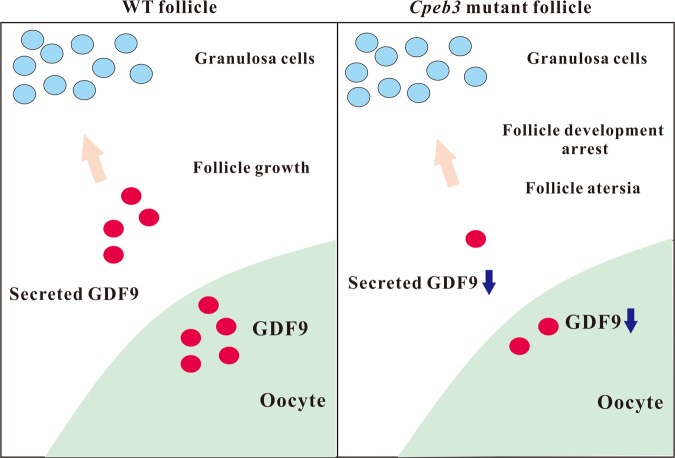
Model of GDF9 regulation by CPEB3 within follicles. Model depicting how CPEB3 might play a role in the regulation of mouse folliculogenesis and follicular atresia.

We have schematically illustrated in Fig. 7 how CPEB3 influences follicle development and atresia. In brief, the illustration shows that CPEB3 is mainly expressed in the oocytes of follicles. The expression pattern of CPEB3 indicates that CPEB3 might exert its role in the developmental stages of folliculogenesis. *Cpeb3* mutation decreased *Gdf9* expression in oocytes, induced granulosa cell apoptosis, and decreased proliferation. These events, in turn, induce the follicles to undergo atresia or inhibit development. Our results furthermore clarify the role of *Cpeb3* in ovarian physiology and pathology and also extend our understanding of potential genes associated with POI in human patients.

## Materials and methods

### Mice

*Cpeb3*-mutant mice were generated by CRISPR/Cas9 (Supplementary Fig. S1A, B, and C) [12]. *Cpeb3* systemic mutation mice with ORF were destroyed. Transgenic mouse lines have C57BL/6 J genomic background. Mice were kept in a well-controlled specific pathogen-free facility with 40–60% humidity, 25 °C constant temperature, and a 12 h light/dark cycle. The animal experiments were approved by the local ethics committee of Harbin Medical University.

### Antibodies

Rabbit monoclonal anti-Beclin1 antibody was purchased from Abcam (ab210498). Mouse monoclonal anti-DDX4/MVH antibody was purchased from Abcam (ab27591). Rabbit polyclonal anti-GDF9 antibody was purchased from Abcam (ab93892). Rabbit monoclonal anti-Cleaved-Caspase3 and anti-PCNA antibodies were purchased from Cell Signaling Technology (9664,13110). Rabbit polyclonal anti-Phospho-Histone H3 antibody was purchased from Cell Signaling Technology (9701). Horseradish peroxidase (HRP)-conjugated secondary antibodies and fluorescein isothiocyanate-conjugated goat anti-rabbit IgG (H+L) were purchased from Zhongshan Golden Bridge Biotechnology Co., LTD (Beijing, China).

### Fertility assay

*Cpeb3*-mutant and WT female 3-month-old mice were housed with WT C57BL/6 J males (2–3 months old), which were proved to have normal fecundity. Copulatory plugs were monitored daily, and plugged females with visibly growing abdomen were moved to separate cages to monitor their pregnancy. The mating process lasted for 6 months. One female mouse was housed with one adult male mouse. The male mice were rotated weekly. Mating cages were monitored daily. The number of pups (both alive and dead) were counted on the first day of delivery.

### Serum hormone measurement

Serum levels of FSH were measured using a mouse FSH ELISA kit (Westang Biotechnology, Shanghai, China). Serum E2 was measured using the E2 ELISA kit (JianCheng Bioengineering Institute, Nanjing, China). All measurements were taken according to the manufacturer’s instructions.

### RNA isolation and real-time PCR

Total RNAs from either ovaries or isolated oocytes were extracted by using Trizol Reagent (Invitrogen, 15596026). The cDNAs were synthesized by using MultiScribe™ Reverse Transcription System (Thermo Fisher, 4311235). Quantitative real-time PCR reactions for target genes were performed using SYBR Green Real-time PCR Master Mix (Applied Biosystems, USA). All experiments were performed in triplicates, and mRNA expression was analyzed using the 2^−ΔΔCT^ calculation method after normalizing with glyceraldehyde-3-phosphate dehydrogenase (housekeeping gene) expression. Primers are shown in Supplementary Table S1.

### Western blotting

The details for conducting western blot as previously described [13]; however, 30–80 μg protein was loaded in polyacrylamide gel (7.5–12.5% conc.), followed by electrophoresis and transfer. The primary and HRP-linked secondary antibodies used in this study are described before.

### TUNEL assay

Terminal deoxynucleotidyl transferase nick end labeling (TUNEL) assays were performed to evaluate cell apoptosis of follicles using 5 μm paraffin-embedded sections, according to the manufacturer’s instructions of In Situ Cell Death Detection Kit, POD (Roche, Cat. No.11684817910).

### Histology

In brief, 3-week, 1-, 3-, and 9-month-old WT and *Cpeb3*-mutant ovaries were fixed in 4% paraformaldehyde at 4 °C for 24 h. The specimens were then dehydrated, cleared in xylene, and embedded in paraffin wax. The embedded specimens were serially sectioned at 5 μm using a rotary microtome (Leica, Frankfurt, Germany). The sections were either stained with hematoxylin and eosin, PAS reaction, or Masson’s trichrome dyes and IHC. The PAS and Masson staining were used to reveal the presence of atretic follicles in the ovarian sections. Primary and secondary antibodies were used in IHC and the stained histological sections were photographed using an inverted microscope.

### Classification of developing follicles in ovarian sections

The follicles in the ovarian histological sections were organized developmentally according to their morphology as primordial, primary, secondary, early antral, and antral follicles. An oocyte surrounded by a single layer of squamous granulosa cells was classified as a primordial follicle and an oocyte surrounded by a single or several layers of cuboidal granulosa cells was classified as a primary or secondary follicle, respectively. When an antrum was present, it was described as an early antral and antral follicle. The presence of the zona pellucida remnants was classified as an end-stage atretic follicle. Every fifth and sixth histological section was selected for comparison and evaluation. Follicles were only counted if they appeared in one histological section but not in the other [32].

### Superovulation and collection of oocytes

Three-month-old female mice were injected i.p. with 10 IU human chorionic gonadotropin (hCG), 48 h after injection of 10 IU PMSG. Mice were killed 13 h after hCG injection. MII eggs and cumulus cells complex were collected from oviducts. After hyaluronidase (1 mg/ml, Sigma, H4272) treatment, the cumulus mass was dissociated and MII eggs were collected in M16 medium. The number of oocytes was recorded by a stereomicroscope.

### Statistical analysis

All data were presented as mean ± SD and performed in triplicates at least. Differences between two groups were compared by a two-tailed Student’s *t* test. Statistical analysis was performed by GraphPad Prism 8.0. *P* value <0.05 was considered statistically significant. *P < 0.05, **P < 0.01, ***P < 0.001, ****P < 0.0001, ns. *P* > 0.05.

## Supplementary information

supplementary legend

Supplementary Table S1

Supplemental Fig. 1

Supplemental Fig. 2

Supplemental Fig. 3
