# Supplementary material for: CPEB3 deficiency in mice affect ovarian follicle development and causes premature ovarian insufficiency
Source: Cell Death Dis. 2021 Dec 20;13(1):21. doi: 10.1038/s41419-021-04374-4 (PMC8688431; doi:10.1038/s41419-021-04374-4)
Supplement: Supplementary file 1 — supplementary legend [file 41419_2021_4374_MOESM1_ESM.doc]

**Figure S1. Construction and verification of *Cpeb3* mutant mice**

1. The design of mice *Cpeb3* specific sgRNA. The sgRNA was designed to target the second exon of the mouse *Cpeb3* gene, the targeting site resides in the 5’ proximal region of the *Cpeb3* open reading frame to achieve the higher mutant efficiency. (B) Verification of gene editing in F0 generation by T7E1 assay. The cleaved bands indicating the successful introduction of mutations at the sgRNA targeted site, as marked by the red arrow. (C) The genotype of wild-type and *Cpeb3* mutant mice. (D) DNA sequencing of WT and *Cpeb3* mutant mice. (E) Total protein extracted from ovaries, testis and brains were loaded and Western blot tests were performed. GAPDH was used as the internal control.

**Figure S2. Histology of WT and *Cpeb3* mutant ovaries.**

(A) CPEB3 antibody was applied in IHC method to manifest the expression pattern in WT mice ovaries. (B) Representative histological appearance of 9 days, 3-week, 1- and 3- and 9-month-old of WT and *Cpeb3* mutant ovaries stained with hematoxylin and eosin. (C) HE staining of WT and *Cpeb3* mutant mice ovaries. Yellow arrowhead indicates oocyte degenerating or developing arrest follicle.Scale bar=20 and 50μm in A; 100μm in B; 50μm in C.

**Figure S3. Immunohistochemistry of WT and *Cpeb3* mutant ovaries.**

(A) Representative micrographs of follicles stained for Cleaved-Capase3 at 1-month-old ovaries. (B) Autophagy levels of ovaries were assessed by IHC with Beclin1 antibody at 1- and 3-month-old ovaries. (C-D) Ovarian sections stained for PCNA and pHIS3 at 1-month-old ovaries. Scale bar=100μm and 50μm in A-D. Data were represented as the mean±SD, n=6. *****P* <0.0001.
