# Supplementary material for: CPEB3 deficiency in mice affect ovarian follicle development and causes premature ovarian insufficiency
Source: Cell Death Dis. 2021 Dec 20;13(1):21. doi: 10.1038/s41419-021-04374-4 (PMC8688431; doi:10.1038/s41419-021-04374-4)
Supplement: Supplementary file 2 — Supplementary Table S1 [file 41419_2021_4374_MOESM2_ESM.doc]

**Table S1. Primers uesd in PCR and qRT-PCR analysis.**

| Primers | Sequence |
| --- | --- |
| Mouse *Cpeb3*-F | 5’-CCCAAGCCCGAAGACAGTAG-3’ |
| Mouse *Cpeb3*-R | 5’-GGAAGAAGCTGTCCTCCA CC-3’ |
| Mouse *Gdf9*-F | 5’-AGCAACCAGGTGACAGGACC-3’ |
| Mouse *Gdf9*-R | 5’-AGGTCACAGTGGAGGAGGAA-3’ |
| Mouse *Foxl2*-F | 5’-AACACCGGAGAAACCAGACC-3’ |
| Mouse *Foxl2*-R | 5’-CGTAGAACGGGAACTTGGCT-3’ |
| Mouse *Kitl*-F | 5’-AGTGACTGTGTGCTCTCTTCA-3’ |
| Mouse *Kitl*-R | 5’-GCCTTTGCGGCTTTCCTATT-3’ |
| Mouse *Cyp11a*-F | 5’-TTCCCCTGGCGACAATGGTT-3’ |
| Mouse *Cyp11a*-R | 5’-AGTGCCCAGCTTCTCCCTGTA-3’ |
| Mouse *Cyp17a*-F | 5’-CAATGACCGGACTCACCTCC-3’ |
| Mouse *Cyp17a*-R | 5’-GGCAAACTCTCCAATGCTGG-3’ |
| Mouse *Fshr*-F | 5’-CGGAACGCCATTGAACTGAGA-3’ |
| Mouse *Fshr*-R | 5’-GGTTGGAGAACACATCTGCCT-3’ |
| Mouse *Gapdh*-F | 5’-TGGCCTTCCGTGTTCCTAC-3’ |
| Mouse *Gapdh*-R | 5’-GAGTTGCTGTTGAAGTCGCA-3’ |
| Mouse *Amh*-F | 5’-TGAAGTGATATGGGAGCCCGA-3’ |
| Mouse *Amh*-R | 5’-AGTAGGGCAGAGGTTCTGTGT-3’ |
| Mouse *Inhibin b*-F | 5’-TCAGCTTTGCAGAGACAGATGG-3’ |
| Mouse *Inhibin b*-R | 5’-ACACCTTGACCCGTACCTTC-3’ |
