# Supplementary figures and images for: CPEB3 deficiency in mice affect ovarian follicle development and causes premature ovarian insufficiency
Source: Cell Death Dis. 2021 Dec 20;13(1):21. doi: 10.1038/s41419-021-04374-4 (PMC8688431; doi:10.1038/s41419-021-04374-4)

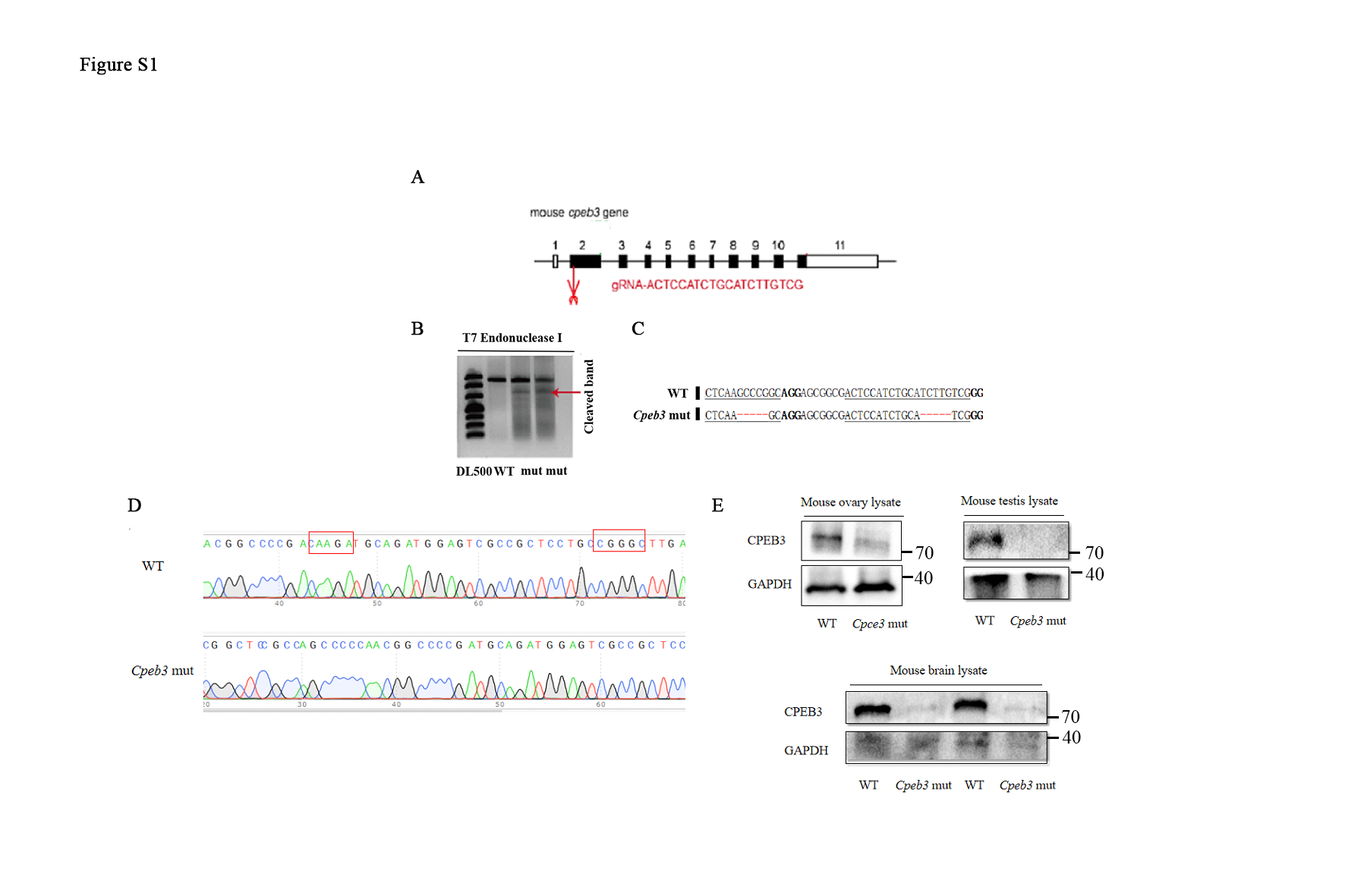

Supplement: Supplementary file 3 — Supplemental Fig. 1 [file 41419_2021_4374_MOESM3_ESM.png]

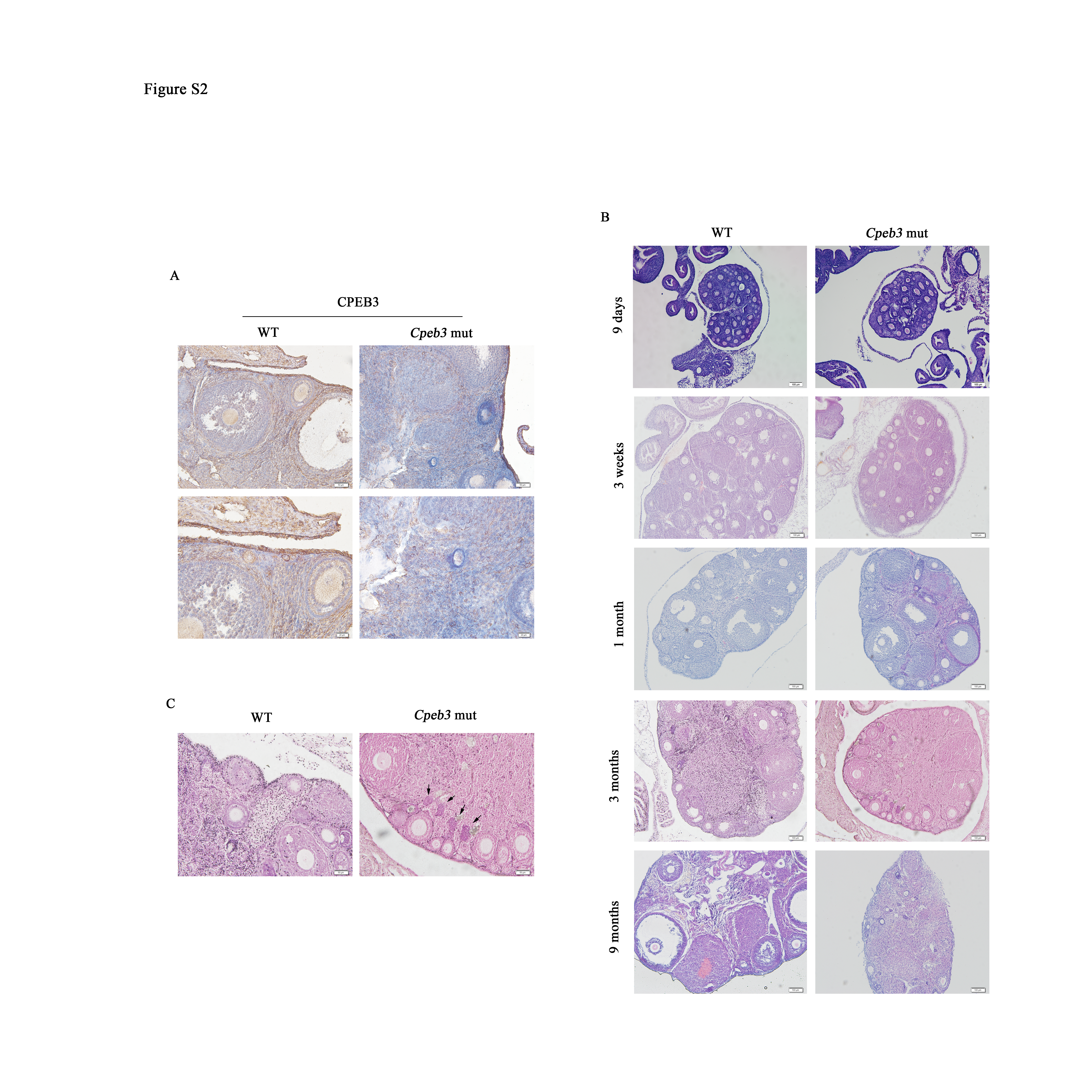

Supplement: Supplementary file 4 — Supplemental Fig. 2 [file 41419_2021_4374_MOESM4_ESM.png]

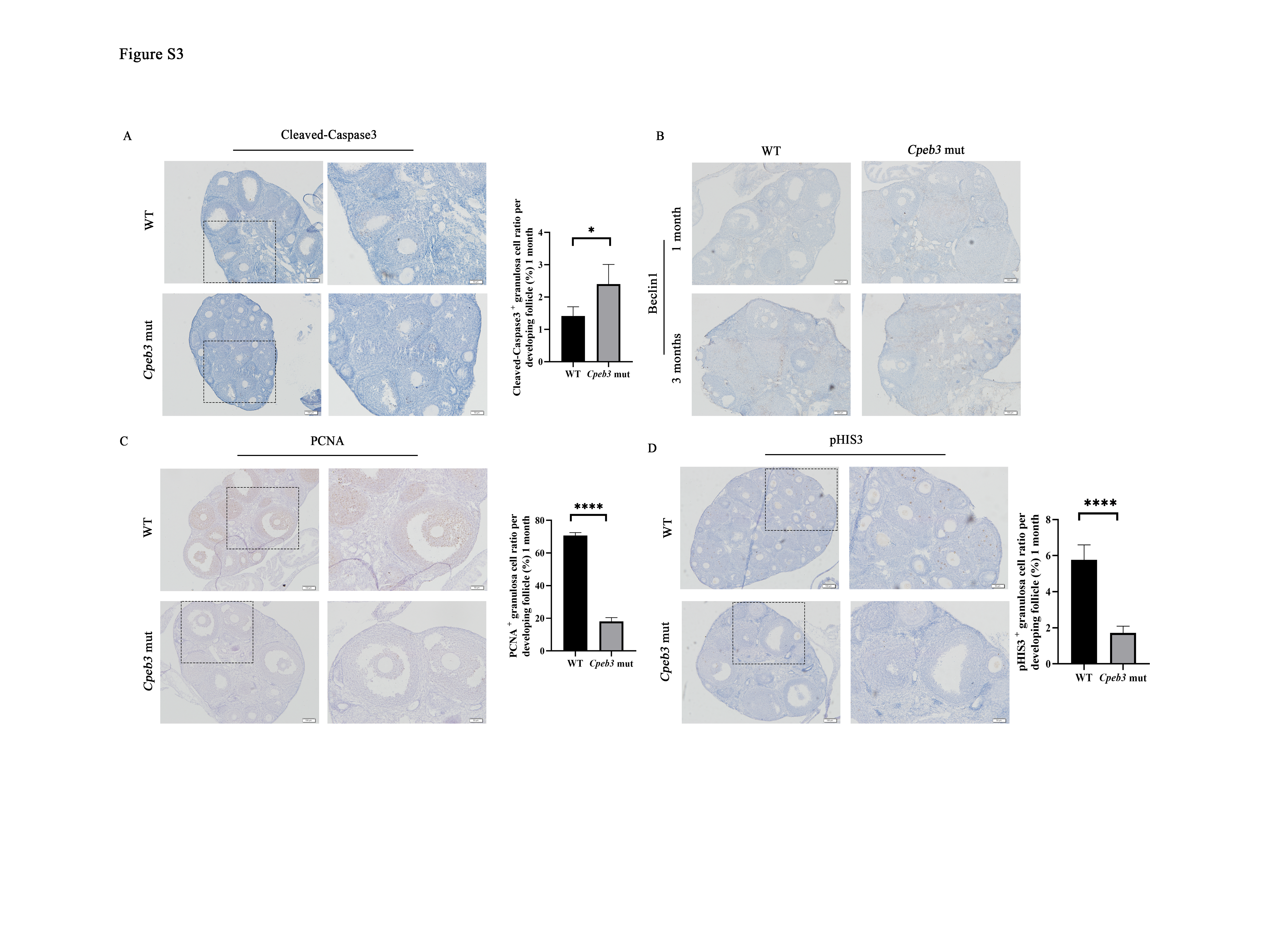

Supplement: Supplementary file 5 — Supplemental Fig. 3 [file 41419_2021_4374_MOESM5_ESM.png]
